# Risk factors for antimony treatment failure in American Cutaneous Leishmaniasis in Northwestern-Argentina

**DOI:** 10.1371/journal.pntd.0009003

**Published:** 2021-01-26

**Authors:** María F. García-Bustos, Gabriela González-Prieto, Alberto E. Paniz-Mondolfi, Cecilia Parodi, Josefina Beckar, Sibila Monroig, Federico Ramos, María C. Mora, Lourdes A. Delgado-Noguera, Yoshihisa Hashiguchi, Daniela Jaime, Sonia Moreno, Luisa Ruiz-Morales, César G. Lemir, Alejandra Barrio

**Affiliations:** 1 Instituto de Patología Experimental, Consejo Nacional de Investigaciones Científicas y Técnicas, Salta, Argentina; 2 Escuela Universitaria en Ciencias de la Salud, Universidad Católica de Salta, Salta, Argentina; 3 Facultad de Ciencias de la Salud, Universidad Nacional de Salta, Salta, Argentina; 4 Instituto de Investigaciones Biomédicas IDB, Departamento de Enfermedades Infecciosas y Medicina Tropical, Laboratorio de Patología de Enfermedades Infecciosas, Clínica IDB Cabudare, Cabudare, Venezuela; 5 Servicio de Otorrinolaringología, Hospital San Bernardo, Salta, Argentina; 6 Servicio de Otorrinolaringología, Hospital Papa Francisco, Salta, Argentina; 7 Leishmania Collaborative Network, Emerging Pathogens Division, The Venezuelan Science Incubator, Cabudare, Venezuela; 8 Decanato de Ciencias de la Salud, Universidad Centroccidental Lisandro Alvarado (UCLA), Barquisimeto, Venezuela; 9 Department of Parasitology, Kochi Medical School, Kochi University, Nankoku, Kochi, Japan; 10 Servicio de Dermatología, Hospital Joaquín Castellanos, Güemes, Salta, Argentina; 11 Servicio de Dermatología, Hospital Señor del Milagro, Salta, Argentina; 12 Servicio de Dermatología, Hospital San Bernardo, Salta, Argentina; 13 Servicio de Infectología, Hospital San Bernardo, Salta, Argentina; National Institutes of Health, UNITED STATES

## Abstract

Background. To date, there is no specific literature available on the determinants for therapeutic failure (TF) with meglumine antimoniate (MA) in Northwestern-Argentina. This study aimed to identify epidemiological, clinical, and treatment-related factors that could be involved in TF. Methodology/Principal Findings. We performed a case-control study. Cases were represented by patients who showed TF after administration of the first course of MA treatment, whereas, controls were determined as patients who evolved towards healing after the first MA cycle received. Crude Odds Ratios and their corresponding 90% confidence intervals (CI) were calculated, and risk factors were then tested by multivariate analysis using logistic binary regression. Three hundred and eighty-four patients with a presumptive diagnosis of ACL were recruited, and 153 with a positive diagnosis were selected. We included in the study 71 patients, who underwent specific treatment with MA, presented complete data on response to treatment, and had a minimum post-treatment follow-up of 6 months in cutaneous leishmaniasis, and 12 months in mucosal leishmaniasis. Of these, 34 (47.9%) presented TF. In the initial analysis, TF was significantly associated with the geographical area of disease acquisition (p = 0.036), the presence of mucosal lesions (p = 0.042), the presence of concomitant skin and mucosal lesions (p = 0.002), and lesion age ≥ 6 months (p = 0.018). Risk factors influencing TF in the final multivariate model included the geographical area where the disease was acquired (adjusted Odd Ratio 8.062; 95% CI 1.914–33.959; p = 0.004), and lesion age ≥ 6 months (adjusted Odd Ratio 10.037; 95% CI 1.383–72.843; p = 0.023). Conclusions/Significance. The results of the present study suggest the existence of some risk factors linked to TF in Northwestern-Argentina, which deserve further investigation. Herein we recorded a high percentage of TF and we described clinical and epidemiological characteristics associated with TF that could be taken into account improving the clinical management of patients.

## Author summary

Cutaneous leishmaniasis and mucosal leishmaniasis are parasitic diseases characterized by the involvement of skin and mucous membranes, respectively. The first-line drug for the treatment is meglumine antimoniate, but its use presents several limitations, such as an aggressive administration schedule, a wide range of local and systemic side effects, great variability in therapeutic response, and an important number of failed treatments. In this study, we explored epidemiological, clinical, and treatment-related factors that could be involved in treatment failure (TF). We included in the study 71 adult subjects who underwent the first cycle of specific treatment with meglumine antimoniate, and we found that the geographical area where the disease was acquired (Yungas ecoregion), and lesion age ≥ 6 months accounted as risk factors for TF. Knowledge of the high percentage of TF recorded, as well as a description of distinct characteristics associated with these failures, could help to improve the clinical management of patients.

## Introduction

The leishmaniases are a group of diseases caused by parasites of the genus *Leishmania*, which are transmitted to humans through the bite of infected female phlebotomine sandflies. The disease in all its clinical expressions is endemic in 98 countries [1], affecting mainly poor populations of the tropical and subtropical belt worldwide. Social, environmental and climatologic factors directly influence the epidemiology of the disease [2]. Over one billion people today live in endemic areas at risk of infection [3], and an estimated 900.000–1.6 million new cases and 20.000 to 40.000 deaths occur annually [1]. Depending on the infecting *Leishmania* species and the immune response of the host, leishmaniases can exhibit a disease spectrum which includes visceral leishmaniasis or "kala-azar", affecting mainly the mononuclear phagocytic system; as well as cutaneous (cutaneous leishmaniasis, CL) and mucosal forms (mucosal leishmaniasis, ML), which are characterized by involvement of skin and mucous membranes, respectively [4].

In the Americas, leishmaniasis is endemic in eighteen countries, from southern United States to northern-Argentina. CL and ML forms, also referred as American Cutaneous Leishmaniasis (ACL), are endemic in ten provinces of northern-Argentina, where they represent a serious public health problem [5]. Orán and San Martín departments (Salta province) located in Northwestern-Argentina (NWA), reported 53.1% of the total number of ACL cases recorded in the country. Both departments are considered hyperendemic areas. Exposure to the sandflies vectors has been positively associated with recent ecological disturbance (deforestation), and with periurban vegetation [6]. Rural workers of low socioeconomic condition, are the most affected populations. *Leishmania (Viannia) braziliensis*, *L (V*.*) guyanensis*, and *L*. *(Leishmania) amazonensis* are commonly isolated from human ACL cases although *L*. *(V*.*) braziliensis* is the main agent associated with ACL outbreaks and subsequent ML cases. We performed the parasitological and molecular diagnosis (kDNA-PCR) of leishmaniasis for many years in collaboration with hospitals of Salta (Argentina) [7]. Most of the patients examined were from Orán and San Martín departments, Salta. However, in a recent study, we found that about a third of the patients from Salta province acquired the disease outside sites of the hyperendemic area [5]. These sites were situated in Chaco-region (Dry-Chaco), and Yungas-region including the department of Chicoana (Salta). Among the patients from these sites, we observed meglumine antimoniate (MA) treatment failure (TF)-cases and a high rate of extensive/severe ML forms [5]. The data of Salta Ministry of Health suggests that the relative number of ML cases has increased, from 3% to 27% during the period 2002–2012. Salomon et al. [8] explain that the decrease of incident cutaneous cases together with the past cutaneous epidemics increased the ratio of mucosal to cutaneous cases. However, this implies that these patients were not treated or they had TF [5].

Currently, the World Health Organization and National Argentinian guidelines for the control of leishmaniasis do not recommend vector control campaigns, as they are not cost-effective. At present early detection of ACL is the main prophylactic measure. Therefore, prevention relies mainly on rapid diagnosis and early treatment [9].

Chemotherapy remains the major control strategy for this group of diseases with MA enduring as the first-line treatment for all clinical forms of ACL [10]. However, its use in the clinical setting exhibits several limitations, given that patients require an aggressive administration schedule (one or two intramuscular daily injections for 21 to 35 days), and often exhibit a wide range of local and systemic side effects [9]. Typical side effects include cardiac, pancreatic, renal and hepatic toxicity, among others [9,11], while demonstrating great variability in therapeutic response, and an important number of failed treatments [12]. Reported data from the literature, mostly on the cutaneous forms of the disease are very variable and show that the proportion of patients who respond to antimonial compounds ranges from 26% to 100% [13]. In previous work, we found that of all ACL cases caused by *Leishmania* (*Viannia*) *braziliensis* in the province of Salta (Argentina), 21% exhibited antimony TF [14].

Many reports on the influence of clinical and epidemiological factors in response to MA treatment in Latin America [13,15–17] are available. In Brazil, Rodrigues *et al*. found that TF was associated with the appearance of 3 or more cutaneous lesions, reports of previous leishmaniasis treatment, body weight above 68 kg, and incomplete treatment schedule [13]. In Peru, Llanos-Cuentas *et al*. [15] and Valencia *et al*. [16] identified young age, permanence of <72 months in the area of disease acquisition, duration of disease <5 weeks, presence of an additional lesion, presence of concomitant distant lesions, infection with *L*. (*V*.) *peruviana*, and infection with *L*. (*V*.) *braziliensis* as risk factors for TF. In Colombia, Castro *et al*. [17] found that TF was associated with age <8 years, disease duration <1 month, regional lymphadenopathy, treatment with MA, and adherence <90%. However, for our region (NWA) there are no specific reports on the potential determinants of TF for this drug. Additionally, the ever-increasing reports of patients with mucosal involvement in the region [5], and the known fact that mucosal forms usually follow an insidious course after the appearance of a primary refractory lesion prompted us to identify epidemiological, clinical, and treatment-related factors that could be involved in TF. We hypothesize that some epidemiological and clinical characteristics of patients with ACL from the province of Salta and surroundings (mainly from NWA), as well as certain characteristics of the treatment scheme with MA administered, constitute risk factors for the appearance of TF.

## Methods

### Ethics statement

The study was conducted according to the principles expressed in the Declaration of Helsinki and approved by the Ethical Committee of Medical College of Salta province, Argentina (Internal Register number 16.032). All adult subjects provided informed written consent, and a parent or guardian child participants provided consent on their behalf.

### Study design

We adopted an observational, case-control design. The reporting of this study conforms to the STROBE statement. (S1 STROBE Checklist). Cases were represented by patients who showed TF after administration of the first course of MA treatment, whereas the controls were patients treated, and who evolved towards healing after the first treatment cycle received.

### Context

The study was carried out at the Microbiology Laboratory, Faculty of Health Sciences, National University of Salta. Patients who attended to this laboratory were admitted in Salta to undergo *Leishmania* diagnosis, and mostly came from high-risk endemic areas of transmission within Salta and adjacent provinces.

### Participants

All patients with a positive diagnosis of ACL (at least one positive result by direct parasitological examination (smear and/or culture) [9], or PCR for *Leishmania* spp. [7], who underwent a first specific cycle with MA, with a post-treatment follow-up of at least 6 months for CL, or 12 months for ML, and referred to the Microbiology Laboratory for *Leishmania* diagnosis between January 2000 and December 2017, were included in the study. The post-treatment follow-up was performed citing the patients immediately (within the first 15 days) and 30 days after finishing the administration of their treatment. We also cited the patients and performed the follow-up 3, 6, 9, and 12 months after the finalization of treatment. Patients diagnosed on clinical grounds alone and/or by intradermal–Montenegro–skin test (MST) who did not undergo specific treatment with MA and who presented a shorter follow-up of less than six months in CL, or less than 12 months in ML or without post-treatment follow-up, were excluded of the study. For *Leishmania* species identification, polymorphism-specific PCR (PS-PCR) was performed, as described in a previous publication of the group [14].

### Variables

#### Impact variable (response to treatment)

For CL, TF was considered if after three months post-treatment there was the presence of one of the following criteria: A. The lesion progressed 50% or more in size, or tested positive for parasites; B. The lesion progressed after having previously regressed; C. The lesion did not completely re-epithelize and/or exhibited persistent inflammatory signs (edema, erythema); D. A new parasitologically positive lesion appeared in the site of a previously healed lesion, or a satellite lesion around the initial one (without suspicion or evidence of reinfection), or there was development of inflammatory signs, with or without ulceration, on a previously healed lesion [16]. Healing was considered if there was the presence of one of the following criteria: A. Complete cure (before 3 months post-treatment) of all lesions (complete epithelialization, absence of inflammatory signs, no evidence of new lesions and complete regression of adenomegaly and lymphangitis); B. Absence of TF criteria [16]. For ML, assessment on the effectiveness of treatment was based on a severity score for mucous lesions, as previously described [18]. Patients who achieved and maintained an improvement of 90% in relation to their initial score during a 12 month-period after completion of treatment were considered cured. Those patients, who did not achieve clinical resolution at 6 months post-therapy, were recorded as TF.

#### Predictive variables

A. Epidemiological and clinical characteristics (sex, age, possible geographical area of acquisition of the disease, type of exposure related to the transmission, clinical form, number and localization of cutaneous lesions, the severity of mucosal lesions, lesions age, *Leishmania* species involved, Chagas disease serological status, presence of secondary bacterial or fungal infections, and presence of other infectious comorbidities. B. Characteristics of the administered treatment, according to the PAHO recommendations for the treatment of ACL [10] (complete or incomplete therapeutic scheme, sufficient or insufficient dose, recommended or less duration, and interruptions of more than two consecutive days). Administration of less than 90% of the prescribed dose was considered insufficient [13]. The therapeutic scheme was considered incomplete when the dose was insufficient; the duration was less than recommended, and if any interruptions were recorded.

### Sources of data

The primary data source was a semi-structured questionnaire, designed to collect data from patients referred for diagnosis and routinely used by the Microbiology Laboratory. Questionnaire included personal, demographic and epidemiological data (place and circumstances where the disease was contracted), relevant clinical information (chief complaint, past medical history related to current disease, other pertinent clinical data, and physical examination). Ancillary testing results to screen for associated infections and their causative agents, as well as clinical data on concomitant pathologies, especially differential diagnoses for ACL were also included. Other data sources included relevant information from clinical charts from other care centers. This information was reviewed in order to complete the data concerning treatment specifications and follow-up.

### Statistical methods

Frequencies and proportions were used to describe categorical variables. For numerical variables, means, medians, standard deviations, and/or ranges were obtained (according to the type of underlying distribution using the Shapiro-Wilk Normality Test). To establish possible associations between the impact variable (response to treatment) and the different predictive variables, comparisons were made using Pearson’s Chi-Square and Fisher's Tests. To compare a numerical variable (age, lesion age) among the studied groups, the Unpaired Student's “T” Test (for normal distributions) and Mann-Whitney's “U” Test (for data that don't pass the normality test) were used for independent samples. Crude Odds Ratios (cOR) and their corresponding 90% confidence intervals (CI) were calculated for each dichotomous nominal variable with significance level ≤ 0.10, using simple logistic regression (SLR). Then these variables were tested by multivariate (MV) analysis through logistic binary regression (LBR) using the “intro” method and obtaining Adjusted OR (aOR). An alpha of 0.10 was chosen for SLR analysis to have potential broader inclusion of variables into the final MV model. Potential confounders were controlled in the analysis phase, through their inclusion in the MV regression. Subgroup analysis was performed in patients with CL and ML.

The predictability of the final model (values of model specificity and sensitivity, and global percentage), and the Hosmer-Lemeshow Goodness of Fit Test were used for model selection. Missing data were not included in the analyses. The final level of significance for LBR was set at p≤ 0.05. The statistical programs GraphPad Prism 6 and SPSS 21 were used.

### Geolocation

Geographical location and distribution of cases (latitude and longitude) was obtained using Latlong [19]. Spatial distribution was complemented with environmental interpolations using the most updated ecoregional landscape database [20], which served as a base layer for smart mapping. Collected data on the geographical location of cases and the distribution of different *Leishmania* species (*L*. *amazonensis* and *L*. *braziliensis*) was mapped using GIS software [21] and plotted for visual inspection onto high-resolution satellite maps. In addition, relative density analysis was assessed employing Heat map symbology (Kernel Density Estimation) to represent point distribution using GIS software [21].

### Registration

The study was registered in the National Registry of Health Research, (Ministry of Health, Argentina), number IS000822 [22].

## Results

**Fig 1:**
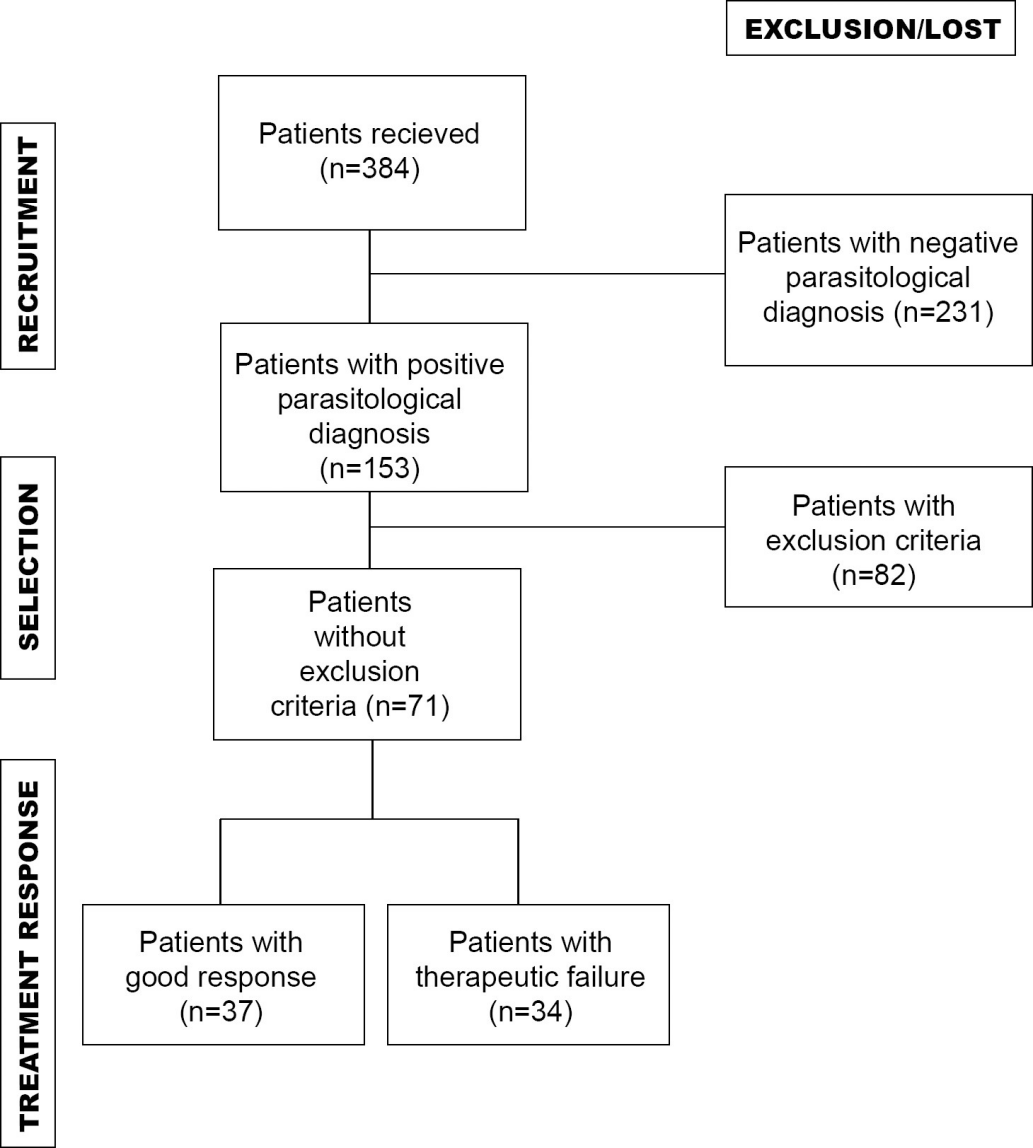
Flowchart of selection and response to treatment among evaluated patients.

Of 384 patients with a presumptive diagnosis of ACL recruited from January 2000 to December 2017, 153 patients (39.8%) had a positive leishmaniasis diagnosis. We included in the study 46.4% of the positive patients (71/153), who underwent specific treatment with MA, and presented complete data on response to treatment. The selected patients also had a minimum post-treatment follow-up of 6 months in CL, and 12 months in ML. Of the 71 patients included in the study, 34 (47.9%) presented TF during the first treatment cycle (cases), while the rest of the patients (n = 37; 52.1%) healed and were considered as controls. Fig 1 shows a flowchart of selection and response to treatment among the two groups of evaluated patients (cases and controls). Of 34 patients with TF after the first cycle of treatment, four were lost during follow up, and 30 underwent a subsequent cycle of treatment. Fourteen patients received alternate therapy with second and third-line drugs. Of the (16) patients who received the second cycle of MA, 68.75% (11/16) relapsed. All the patients who received an incomplete second cycle of MA treatment (six individuals) presented TF.

### Clinical, epidemiological and therapeutic characteristics

The clinical and epidemiological features of most individuals who comprise this population of patients were described in a previous publication from our group [5]. The studied characteristics of cases and controls, including details on their treatment regimes in both patients’ groups, are depicted in Table 1. This table also shows associations among studied variables through Pearson's Chi-Squared Test or Fisher's Exact Test for categorical variables, and Unpaired Student's “T” Test or Mann-Whitney's “U” Test (p-value). In this analysis, TF was significantly (p≤ 0.05) associated with (a) geographical area where the disease was presumably acquired (Yungas ecoregion) (p = 0.036), (b) presence of mucosal lesions (p = 0.042), (c) presence of concomitant skin and mucosal lesions (p = 0.002), and (d) lesion age ≥ 6 months (p = 0.018).

**Table 1:** Association between demographic, epidemiological, clinical and therapeutic characteristics of ACL patients and cure/failure outcome after first cycle of MA treatment.

	Treatment outcome		
	Failure	Cure	
Characteristic	n = 34 (47.9%)	n = 37 (52.1%)	*P*
**EPIDEMIOLOGICAL AND CLINICAL CHARACTERISTICS**			
**GENDER**			*0*.*268* ^*a*^
• Male	30 (88.2)	29 (78.4)	
• Female	4 (11.8)	8 (21.6)	
**AGE (years)**			*0*.*634* ^*b*^
Mean (± SD)	38.82 (± 16.87)	40.62 (± 14.77)	
**ECOREGION WHERE THE INFECTION WAS ACQUIRED**			*0*.*036* ^*a*^
• Yungas	24 (70.6)	17 (45.9)	
• Dry Chaco	10 (29.4)	20 (54.1)	
**OCCUPATION/HOUSINGS/ACTIVITIES**			*0*.*208* ^*a*^
• Rural work	24 (70.6)	25 (67.6)	
• Periurban or rural housing	9 (26.5)	7 (18.9)	
• Recreational trips	1 (2.9)	5 (13.5)	
**CLINICAL FORMS**			*0*.*042* ^*a*^
• Cutaneous	12 (35.3)	22 (59.5)	
• Mucosal	22 (64.7)	15 (40.5)	
❖ Concomitant cutaneous and mucosal	8/34 (23.5)	0/37 (0)	*0*.*002* ^*c*^
**CUTANEOUS LESIONS**			
• Localization of single lesions			*0*.*361* ^*a*^
❖ Head	4 (57.1)	4 (25)	
❖ Upper limb	1 (14.3)	6 (37.5)	
❖ Lower limb	2 (28.6)	4 (25)	
❖ Trunk	0 (0)	2 (12.5)	
• Number of lesions			*0*.*459* ^*c*^
❖ 1	7 (58.3)	16 (72.7)	
❖ ≥2	5 (41.7)	6 (27.3)	
**MUCOSAL LESIONS**			
• Severity			*0*.*554* ^*c*^
❖ Mild	1 (4.5)	2 (13.3)	
❖ Extensive	21 (95.5)	13 (86.7)	
**LESION AGE (months)**			
Median (min–max)	12 (1–360)	4 (0.75–120)	*0*.*0123* ^*d*^
Ranges			*0*.*018* ^*a*^
• ≥ 6	21 (61.8)	14 (37.8)	
• < 6	10 (29.4)	22 (59.5)	
• Unknown	3 (8.8)	1 (2.7)	
***Leishmania* spp.**			*0*.*612* ^*c*^
• *L*. *(V*.*) braziliensis*	29 (85.3)	27 (73)	
• *L*. *(L*.*) amazonensis*	1 (2.9)	3 (8.1)	
• Unknown	4 (11.8)	7 (18.9)	
**ANTI-*T*. *cruzi* ANTIBODIES**			*0*.*210* ^*a*^
• Yes	14 (41.2)	6 (16.2)	
• No	14 (41.2)	13 (35.1)	
• Unknown	6 (17.6)	18 (48.7)	
**SECONDARY MICROBIAL INFECTION**			*0*.*464* ^*a*^
• Yes	15 (44.1)	8 (21.6)	
• No	8 (23.5)	7 (18.9)	
• Unknown	11 (32.4)	22 (59.5)	
**CONCOMITANT INFECTIOUS DISEASE**			*0*.*218* ^*a*^
• Yes	15 (44.1)	8 (21.6)	
• No	15 (44.1)	16 (43.2)	
• Unknown	4 (11.8)	13 (35.1)	
**THERAPEUTIC CHARACTERISTICS**			
THERAPEUTIC SCHEME^*^			*0*.*08* ^*a*^
• Incomplete	19 (55.9)	13 (35.1)	
• Complete	15 (44.1)	24 (64.9)	
DOSE^*^			*0*.*201* ^*a*^
• Insufficient	12 (35.3)	8 (21.6)	
• Sufficient	22 (64.7)	29 (78.4)	
DURATION^*^			*0*.*248* ^*c*^
• Less	5 (14.7)	2 (5.4)	
• Recommended	29 (85.3)	35 (94.6)	
INTERRUPTIONS^**^			*0*.*111* ^*a*^
• Yes	11 (32.4)	6 (16.2)	
• No	23 (67.6)	31 (83.8)	

Data are no. (%) of patients, unless otherwise noted. Percentages represent the categories consigned in columns.

* As recommended by PAHO for the treatment of leishmaniasis in the Americas [10].

** Interruptions of more than two consecutive days [13].

a Pearson's Chi-Squared Test

b Unpaired Student's “T” Test

c Fisher's Exact Test

d Mann-Whitney's “U” Test

### Logistic regression

All variables that remained associated with TF at a significance level of 90% (p≤ 0.1) were included in the analysis. First, the calculation of the cOR was performed through bivariate (BV) analysis using SLR; then, the aOR was obtained through MV analysis using LBR (Table 2). Gender and age were also included because of their possible influence in the therapeutic response. One variable with significance in the BV analysis was excluded from the MV model, concomitant skin and mucosal lesions, because of the impossibility of calculating the cOR (too small sample). Potential confounders (mucosal disease and lesion age) were controlled in the analysis phase, through their inclusion in the MV analysis (patients with the mucosal disease present longer time of evolution of the disease than patients with the cutaneous form).

**Table 2:** BV and MV analysis of factors associated with TF in ACL patients.

	Bivariate analysis			Multivariate analysis		
FACTOR	cOR	90% CI	*P*	aOR	95% CI	*P*
Yungas ecoregion	2.824	1.240–6.432	***0*.*038***	8.062	1.914–33.959	***0*.*004***
Lesion age ≥ 6 months	3.300	1.416–7.690	***0*.*020***	10.037	1.383–72.843	***0*.*023***
Incomplete therapeutic scheme	2.338	1.048–5.216	*0*.*082*	4.017	0.761–21.198	*0*.*101*
Age (≥40 years)	0.663	0.302–1.459	*0*.*391*	0.407	0.116–1.429	*0*.*160*
Male gender	2.069	0.692–6.182	*0*.*275*	3.219	0.541–19.568	*0*.*199*
Treatment interruptions	2.471	0.956–6.387	*0*.*117*	0.537	0.085–3.380	*0*.*508*
Mucosal clinical form	2.689	1.199–6.028	*0*.*044*	1.613	0.266–9.788	*0*.*603*

cOR: Crude Odds Ratios. aOR: Adjusted Odds Ratios. CI: confidence intervals.

Four patients were excluded automatically from the final model (because of missing data). Risk factors for TF in the final MV model were the geographical area of exposure/infection (Yungas ecoregion) (p = 0.004) and lesion age ≥ 6 months (p = 0.023). Model specificity and sensitivity values were 75.0 and 67.7, respectively, with a global percentage value of 71.6. Hosmer-Lemeshow Goodness of Fit Test value was 0.826.

In the subgroup analysis, we found that for CL patients, trying to find associations among studied variables, none of them remained significantly associated with TF (p> 0.05). However, the p values for the ecoregion were close to being significant, both in the SLR and LBR (p = 0.06). In patients with ML, the ecoregion variable remained associated with TF in the MV analysis (p = 0.031), and the p-value of the lesions age remained close to significance (p = 0.06).

Table 2 shows the unadjusted ORs (cOR) from the SLR analysis (BV analysis) and the aORs of the LBR (MV analysis) in the complete cohort of patients, with their corresponding CI.

### Geographic distribution of patients

**Fig 2:**
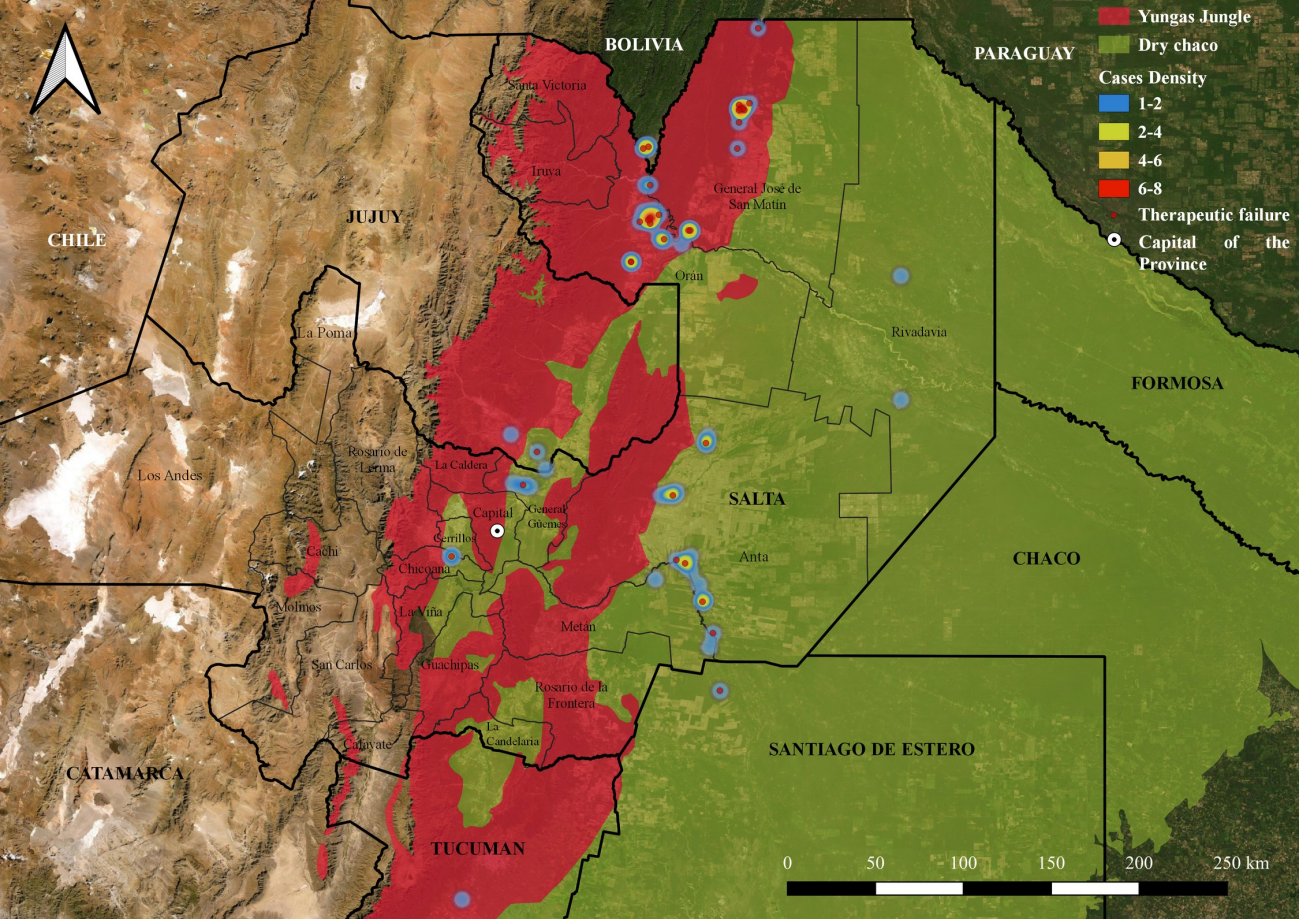
Geographic clustering of patients. Heat map showing the density of patients according to the probable site of infection. The density of patients is shown in a spectrum of blue (1–2 patients) to red (6–8 patients). Also, the map shows the distribution of those patients with TF. GIS software: QGIS 3.14 version, https://www.qgis.org/es/site/; Argentina Republic political division layer: Aeroterra S.A. División política de provincias de la República Argentina. 2007; Ecoregional distribution layer: Brown A, Martinez Ortiz U, Acerbi M, Corcuera J, Pacheco S. Conclusiones de la situación ambiental por ecorregiones. In: Brown A., Martínez Ortiz U., Acerbi JC M., editor. La Situación Ambiental Argentina 2005. Buenos Aires: Fundación Vida Silvestre Argentina; 2006. pp. 373–378.

Geographic clustering of patients occurred evenly throughout the Dry Chaco and Yungas jungle regions (Fig 2). However, the highest density of patients according to the probable site of infection was observed in the Yungas, in Orán and San Martín Departments, both hyper-endemic areas for ACL [5]. Cases of TF are distributed in both ecoregions, but mainly in the Yungas (Fig 2), in accordance with that observed in the MV analysis.

**Fig 3:**
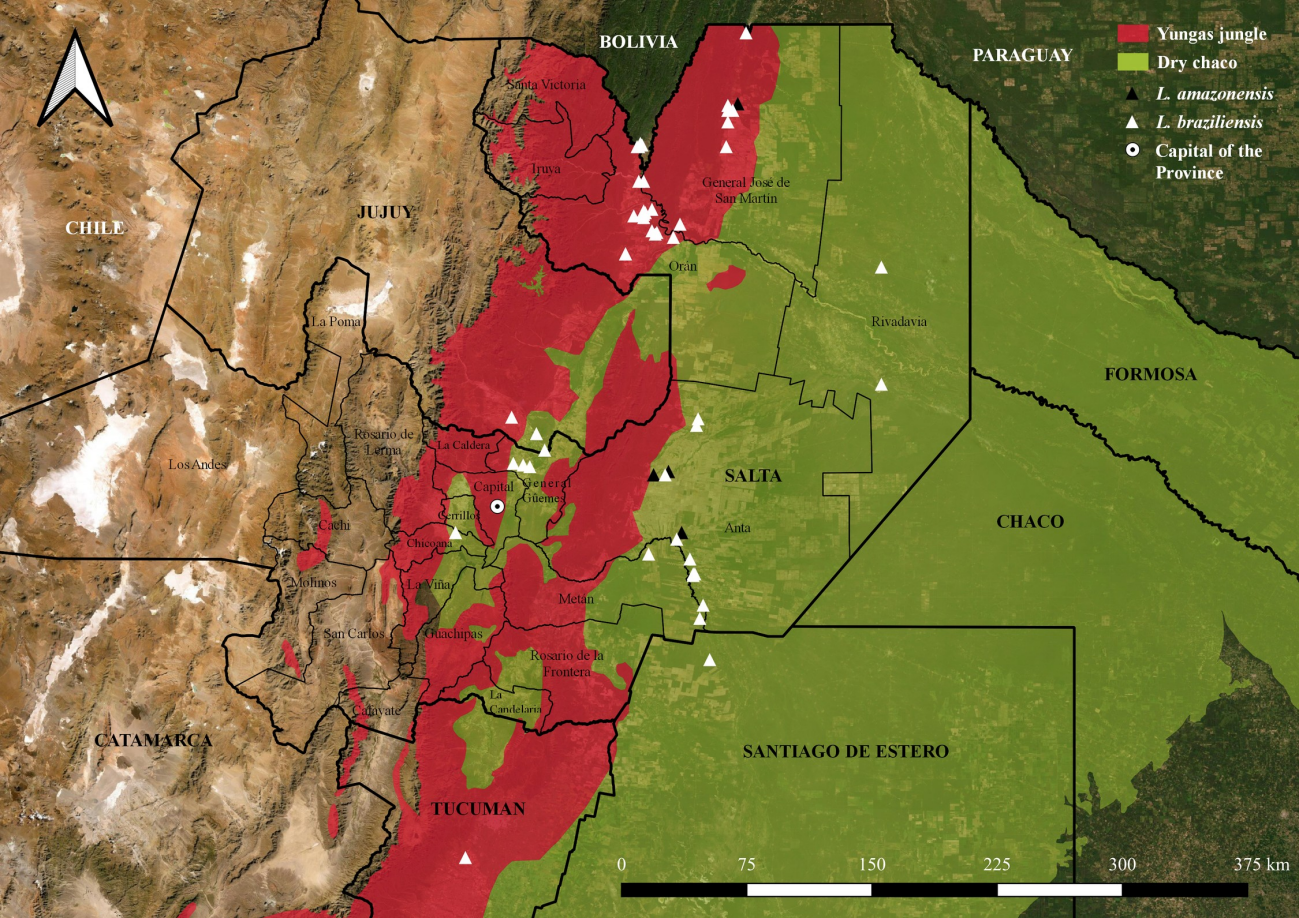
Geographical distribution of patients according to the identified *Leishmania* species. The location of each individual represents the place where the infection was presumably acquired. White triangles: Patients infected with *L*. (*V*.) *braziliensis*. Black triangles: Patients infected with *L*. (*L*.) *amazonensis*. GIS software: QGIS 3.14 version, https://www.qgis.org/es/site/; Argentina Republic political division layer: Aeroterra S.A. División política de provincias de la República Argentina. 2007; Ecoregional distribution layer: Brown A, Martinez Ortiz U, Acerbi M, Corcuera J, Pacheco S. Conclusiones de la situación ambiental por ecorregiones. In: Brown A., Martínez Ortiz U., Acerbi JC M., editor. La Situación Ambiental Argentina 2005. Buenos Aires: Fundación Vida Silvestre Argentina; 2006. pp. 373–378.

Identificated cases of *L*. (*V*.) *braziliensis* presented more commonly in a scattered fashion throughout the jungle (Yungas) and piedmont areas; whereas, *L*. (*L*.) *amazonensis* cases seemed to locate more commonly in the Dry Chaco region (Fig 3).

## Discussion

This study was based on an observational, case-control design to identify epidemiological, clinical, and treatment-related factors that could be involved in MA TF. In this context, a considerable rate (47.9%) of MA TF-cases was found, and the MV analysis model exhibited a significant association of TF with both, the geographical area of exposure/infection (Yungas ecoregion) and lesion age ≥ 6 months, as possible risk factors.

To establish possible associations among studied variables, comparisons were made using Pearson’s Chi-Square Test, Fisher's Test, Unpaired Student's “T” Test and Mann-Whitney's “U” Test. All variables that in these analyses remained associated with TF at a significance level of 90% (p≤ 0.1) (geographical area of exposure/infection, clinical form, lesion age ≥ 6 months, complete or incomplete therapeutic scheme, presence of treatment interruptions) were included in the MV analysis using LBR. Gender and age were also included because of their possible influence in the therapeutic response. Risk factors for TF in the final MV model were the geographical area of exposure/infection (Yungas ecoregion) and lesion age ≥ 6 months, as it was exposed at the beginning. These results were obtained including all patients, without discriminating the clinical form (despite CL and ML are two different clinical syndromes), due to the small size of the sample. When we analyzed these groups separately, in the CL subgroup, the p-value of the ecoregion variable remains close to significance, both in the BV and MV analysis. In the ML subgroup, in the MV analysis, the ecoregion variable remains associated with TF, and the p-value of the lesions age remains close to significance. Although the subgroup analysis reduces the statistical power of the tests, we could affirm that the ecoregion would constitute a risk factor associated with TF, regardless of the clinical form. Also the age of the lesions would constitute a risk factor for TF (longer evolution, increased risk of TF), mainly in the mucous form of the disease.

The precise reason related to the different rates of occurrence of TF-cases in Yungas (70.6%) and Dry-Chaco (29.4%) is not clear. It is however assumed that the difference between the two ecoregions might be caused by factors of parasites and host subjects, such as the difference of strains/genotypes, the susceptibility of the *Leishmania* against MA, and the host immune system (e.g., immunosuppression due to other infectious diseases or malnutrition) [23,24] The intraspecific differences of *Leishmania* species could be responsible for this finding. As most of the parasites isolated in our group of patients correspond to *L*. *braziliensis*, we believe that therapy outcome is highly influenced by the presence of strain variability within species which are distributed differently within transmission areas across the region. Marco et al. [25] found genotypic differences between parasites of *L*. *braziliensis* from two different zones of the province of Salta: "Jungle of transition", located into Yungas Ecoregion, and "Western Chaco", corresponding to Dry Chaco Ecoregion. Also, Locatelli and cols. [26] identified two different genotypes of *L*. *braziliensis* (Ab1 and Ab2), and one of them (Ab2) was only found in the zone of the Zenta valley (Yungas Ecoregion), which is where we found most cases of TF. Studies from Brazil reported intraspecific differences (e.g., genetic, ultrastructural) in isolates from patients with different responses to treatment [27,28].

Regarding the time of lesion evolution, we found that the patients with more than 6 months of disease progression, presented with a higher risk of TF. On this note, in previous work performed by our group, we investigated the evolution of the T cell differentiation profile in ACL, taking into account that early differentiated T cells are considered to confer best protective immunity while highly differentiated T cells present signs of senescence and exhaustion [29,30]. We investigated the evolution of T-cell differentiation phenotypes in relation to illness duration throughout the first year of evolution in localized CL infection. We found that early differentiated CD8+ T cells inversely correlated with illness duration, while late differentiated and terminal effectors CD8+ T cells positively correlated with duration of infection. Besides, the predominance of an early differentiated profile was preferentially found among patients who were able to heal their lesions [31]. In accordance, other studies have reported that the most frequent T-cell population in peripheral CD4+ and CD8+ T cells among recovered CL volunteers were the naïve T cells [32,33]. In addition, we found that patients suffering ML showed the highest differentiated profile in both CD4^+^ and CD8^+^ T cells subpopulations together with the elevation of senescence markers and cytolytic molecules, while CL showed a less pronounced phenotype [31]. The result of our subgroup analysis in relation to the time of evolution of the lesions is in agreement with previous findings.

In contrast to our results, Llanos-Cuentas et al. [15] and Valencia et al. [16] in Peru and Castro et al. in Colombia [17], reported early treatment intervention (evolution time of five weeks or less) as a risk factor associated with TF in CL. They hypothesized that the administration of treatment before reaching an effective acquired immune response is the likely explanation for this phenomenon. However, the authors also comment that these studies examined a high percentage of pediatric patients, which given the variable degree of maturity of their immune status suggest that this age group is particularly vulnerable and further a risk factor for acquiring CL. Conversely, in our population, there was a majority of adult cases with more than one month of evolution at the initiation of treatment, and a high percentage of individuals with the clinical mucosal form.

Concerning initials association analyses between demographic and clinical variables about cure and failure after the first cycle of treatment (Table 1), we found that TF predominated within the mucosal clinical form. In agreement with our previous immunological findings, as it was already exposed, the inability of T cells to control the infection presumably due to exhaustion after persistent activation could also account for the higher rate of TF that we found in mucosal cases [31]. Also, Martínez-Valencia et al. demonstrated a high frequency of *Leishmania* parasites persistence in mucosal tissues after the end of treatment and suggests that mucosal sites could be a privileged niche for parasite persistence after drug treatment, either by pharmacokinetic differences in drug distribution and accumulation and/or immunological divergence between mucosal vs. skin tissues [34].

Interestingly we found that the whole group of patients with concomitant mucous and cutaneous lesions presented TF. There is scarce mention in the literature about this clinical manifestation and its treatment response. Boaventura et al. [35] reported six cases of concomitant early mucosal and cutaneous leishmaniasis in a cohort of 220 patients from Bahía state, Brazil. In contrast to our results, these patients responded adequately to antimonial treatment. This discrepancy in therapeutic response might be due to the prolonged time of evolution of the lesions in our patients. On the other hand, Valencia et. al. described the presence of “concomitant-distant” lesions as a predictor of TF, that could appear due to an intrinsic immune failure favoring metastasis [16].

Analyzing the characteristics of therapeutic regimens administered, we observed that a high percentage of patients who were under a first incomplete treatment presented TF (59.4%), and all the patients who received an incomplete second cycle of treatment presented TF. Moreover, of the 17 patients who presented interruptions in the administration of the first cycle of treatment, 11/17 (64.7%) presented TF. These considerations are in accordance with the cohort studied by Rodrigues et al. [13] in Brazil, and Castro in Colombia [17], and are probably related to the lower drug exposure among this groups of patients. In our cohort, we observed that interruptions mainly occurred in patients who completed treatment on an outpatient basis. Treatment abandonment is very common in isolated communities in the studied area, and others, especially in Latin America. There were also interruptions in cases in which, by medical prescription, the treatment was administered in several cycles of 15 days each, separated by intervals of 7 to 15 days for approximately one year. Before 2005, this was a frequently used schedule in our country, alike other regions [13]. Treatment was also interrupted several times due to the presence of severe toxicity. These findings highlight the importance, first, of following current recommendations for ACL treatment [10], and also of carrying out strict control of the patient’s adherence to treatment. Treatment adherence is a known determinant of clinical response [12,13,17]. If a patient is unable to complete the therapeutic regimen under hospitalization, the possibility of a supervised or directly-observed treatment should be considered. These interventions have shown a positive effect regarding the therapeutic response to antimicrobials [17,36].

Interestingly, we found that 11 out of 16 patients who received the second cycle of antimonial compounds, presented successive TF. Some studies suggested that the association between TF and history of previous treatment might be due, among other factors, to insufficient dosing, promoting the selection of drug-resistant phenotypes to antimonials [12,13,37]. Nevertheless, the hypothesis that low-dose treatments induce selection of resistant parasites is still controversial and has not been confirmed for parasites of the *Viannia* genus [38]. Despite that, we think that the use of high tailored chemotherapy doses or a longer course of treatment with antimonial compounds should be considered during initial cycles [13] to prevent strain selection. Further research to understanding resistance profiles as well as the possible factors influencing the appearance and selection of antimony resistant strains in the region are needed.

Our results are coincident to those published by Rodrigues *et al*. [13] who described an incidence of TF of 47% in a retrospective cohort of CL patients from a geographical area, where *L*. *(V*.*) braziliensis* is the predominant species. Nevertheless, it should be considered that the percentage of TF found in our population, could be overestimating the real situation. The reason for this is that many of the patients that we care for come from remote areas referred to our center for specialized care given their complicated clinical presentations or refractoriness to treatment. Patients with uncomplicated forms are usually managed and followed at local ambulatory centers. Also, after clinical cure, most patients abandon follow up. This point could be considered a limitation of the present study. A systematic approach of random sampling would have probably shown a much more accurate estimate of the true TF rate.

Although the present study is a preliminary analysis, the results point towards the existence of key factors associated with TF in patients from NWA, and how treatment outcome is distributed differently within transmission areas. Further unraveling the potential factors influencing therapy resistance in the region will allow to better understand the ecology of the disease and to design adequate preventive measures.

In Argentina, ACL cases are mostly diagnosed adequately and therefore treated accordingly. However, the factors related to MA TF-cases found in the present ecoregions, Yungas and Dry-Chaco should be investigated precisely, including MA management in the areas. Given the limited resources of antileishmanial drugs available, it is imperative that effective monitoring of their use should be done to prevent the emergence of resistance. In addition to free availability to the drug (which is the case of Argentina) plus directly observed treatment performed to ensure compliance, health education about the disease and the importance of a full treatment-course is essential to control the disease [39].

## Supporting information

S1 STROBE Checklist STROBE checklist. (PDF)
