# Supplementary material for: Risk factors for antimony treatment failure in American Cutaneous Leishmaniasis in Northwestern-Argentina
Source: PLoS Negl Trop Dis. 2021 Jan 26;15(1):e0009003. doi: 10.1371/journal.pntd.0009003 (PMC7864468; doi:10.1371/journal.pntd.0009003)
Supplement: S1 STROBE Checklist — (PDF) [file pntd.0009003.s001.pdf]

STROBE Statement—Checklist of items that should be included in reports of ***case-control studies***

	Item No	Recommendation	Section and paragraph
**Title and abstract**	1	(*a*) Indicate the study's design with a commonly used term in the title or the abstract	Abstract, paragraph 2
		(*b*) Provide in the abstract an informative and balanced summary of what was done and what was found	Abstract, paragraphs 2 and 3
**Introduction**			
Background/rationale	2	Explain the scientific background and rationale for the investigation being reported	Introduction, paragraphs 1-5
Objectives	3	State specific objectives, including any prespecified hypotheses	Introduction, paragraph 5 (lines 151-160)
**Methods**			
Study design	4	Present key elements of study design early in the paper	Methods, paragraph 1 (Study design)
Setting	5	Describe the setting, locations, and relevant dates, including periods of recruitment, exposure, follow-up, and data collection	Methods, paragraphs 2 (Context), 3 (Participants), and 6 (Sources of data)
Participants	6	(*a*) Give the eligibility criteria, and the sources and methods of case ascertainment and control selection. Give the rationale for the choice of cases and controls	Methods, paragraphs 1(Study design), 3 (Participants), 4 (variables: Impact variable [response to treatment]), and 6 (Sources of data)
		(*b*) For matched studies, give matching criteria and the number of controls per case	
Variables	7	Clearly define all outcomes, exposures, predictors, potential confounders, and effect modifiers. Give diagnostic criteria, if applicable	Methods, paragraphs 4 (Impact variable) and 5 (Predictive variables)
Data sources/ measurement	8	For each variable of interest, give sources of data and details of methods of assessment (measurement). Describe comparability of assessment methods if there is more than one group	Methods, paragraphs 4 (Impact variable), 5 (Predictive variables), 6 (Sources of data), and 8 (Geolocation)
Bias	9	Describe any efforts to address potential sources of bias	Methods, paragraph 3 (Participants)
Study size	10	Explain how the study size was arrived at	Methods, paragraphs 2 (Context), and 3 (Participants)
Quantitative variables	11	Explain how quantitative variables were handled in the analyses. If applicable, describe which groupings were chosen and why	Methods, paragraph 7 (Statistical methods)
Statistical methods	12	(*a*) Describe all statistical methods, including those used to control for confounding	Methods, paragraph 7 (Statistical methods)
		(*b*) Describe any methods used to examine subgroups and interactions	Methods, paragraph 7 (Statistical methods)
		(*c*) Explain how missing data were addressed	Methods, paragraph 7 (Statistical methods)
		(*d*) If applicable, explain how matching of cases and	

		controls was addressed	
		(*e*) Describe any sensitivity analyses	
**Results**			
Participants	13*	(a) Report numbers of individuals at each stage of study—eg numbers potentially eligible, examined for eligibility, confirmed eligible, included in the study, completing follow-up, and analysed	Results, paragraph 1 and figure 1
		(b) Give reasons for non-participation at each stage	Results, paragraph 1
		(c) Consider use of a flow diagram	Results, figure 1
Descriptive data	14*	(a) Give characteristics of study participants (eg demographic, clinical, social) and information on exposures and potential confounders	Results, paragraph 2, table 1 and figure 2
		(b) Indicate number of participants with missing data for each variable of interest	Results, paragraph 1 and figure 1
Outcome data	15*	Report numbers in each exposure category, or summary measures of exposure	Results, figure 1 and table 1
Main results	16	(*a*) Give unadjusted estimates and, if applicable, confounder-adjusted estimates and their precision (eg, 95% confidence interval). Make clear which confounders were adjusted for and why they were included	Results, paragraph1-4. Tables 1, 2. Figures 2, 3.
		(*b*) Report category boundaries when continuous variables were categorized	Results, table 1
		(*c*) If relevant, consider translating estimates of relative risk into absolute risk for a meaningful time period	

Other analyses	17	Report other analyses done—eg analyses of subgroups and interactions, and sensitivity analyses	Tables 1 and 2
**Discussion**			
Key results	18	Summarise key results with reference to study objectives	Discussion, paragraph 1
Limitations	19	Discuss limitations of the study, taking into account sources of potential bias or imprecision. Discuss both direction and magnitude of any potential bias	Discussion, paragraphs 2 and 11
Interpretation	20	Give a cautious overall interpretation of results considering objectives, limitations, multiplicity of analyses, results from similar studies, and other relevant evidence	Discussion, after every result, in paragraphs 3 (lines 416-425), 4, 6-10
Generalisability	21	Discuss the generalisability (external validity) of the study results	Discussion, paragraphs 10, 11
**Other information**			
Funding	22	Give the source of funding and the role of the funders for the present study and, if applicable, for the original study on which the present article is based	

*Give information separately for cases and controls.

Note: An Explanation and Elaboration article discusses each checklist item and gives methodological background and published examples of transparent reporting. The STROBE checklist is best used in conjunction with this article (freely available on the Web sites of PLoS Medicine at http://www.plosmedicine.org/, Annals of Internal Medicine at http://www.annals.org/, and Epidemiology at http://www.epidem.com/). Information on the STROBE Initiative is available at http://www.strobe-statement.org.
